# A Role of Canonical Transient Receptor Potential 5 Channel in Neuronal Differentiation from A2B5 Neural Progenitor Cells

**DOI:** 10.1371/journal.pone.0010359

**Published:** 2010-05-07

**Authors:** Hye Young Shin, Yun Hwa Hong, Sung Soo Jang, Hong Gu Chae, Seung Leal Paek, Hyo Eun Moon, Dong Gyu Kim, Jun Kim, Sun Ha Paek, Sang Jeong Kim

**Affiliations:** 1 Department of Neurosurgery, Cancer Research Institute, Ischemic/Hypoxic Disease Institute, College of Medicine, Seoul National University, Seoul, Korea; 2 Department of Physiology, Department of Brain and Cognitive Sciences, Neuroscience Research Institute Medical Research Center, College of Medicine, Seoul National University, Seoul, Korea; 3 Clinical Research Institute, Seoul National University Hospital, Seoul, Korea; University of Sydney, Australia

## Abstract

Store-operated Ca^2+^ entry (SOCE) channels are the main pathway of Ca^2+^ entry in non-excitable cells such as neural progenitor cells (NPCs). However, the role of SOCE channels has not been defined in the neuronal differentiation from NPCs. Here, we show that canonical transient receptor potential channel (TRPC) as SOCE channel influences the induction of the neuronal differentiation of A2B5^+^ NPCs isolated from postnatal-12-day rat cerebrums. The amplitudes of SOCE were significantly higher in neural cells differentiated from proliferating A2B5^+^ NPCs and applications of SOCE blockers, 2-aminoethoxy-diphenylborane (2-APB), and ruthenium red (RR), inhibited their rise of SOCE. Among TRPC subtypes (TRPC1-7), marked expression of TRPC5 and TRPC6 with turned-off TRPC1 expression was observed in neuronal cells differentiated from proliferating A2B5^+^ NPCs. TRPC5 small interfering RNA (siRNA) blocked the neuronal differentiation from A2B5^+^ NPCs and reduced the rise of SOCE. In contrast, TRPC6 siRNA had no significant effect on the neuronal differentiation from A2B5^+^ NPCs. These results indicate that calcium regulation by TRPC5 would play a key role as a switch between proliferation and neuronal differentiation from NPCs.

## Introduction

Cytosolic Ca^2+^ is a ubiquitous second messenger to control a great number of cellular functions ranging from short-term responses such as contraction and secretion to long-term regulation of transcription, growth and cell division as well as development of embryonic cells [1]–[3]. Ca^2+^ entry channel is classified into two types: voltage operated calcium channels (VOCCs) and non-voltage operated calcium channels (non-VOCCs) [4]. Neural progenitor cells (NPCs) have few VOCCs, indicating that non-VOCCs may regulate the differentiation of NPCs [5], [6], and canonical transient receptor potential channel (TRPC) is one of the non-VOCCs [7]. Here, we focused on the physiological function of the TRPCs as store-operated Ca^2+^ entry (SOCE) in the neural differentiation of NPCs.

Based on sequence similarity and function, seven TRPC homologs (TRPC1-7) can be subdivided into four groups: TRPC 4/5 (group 1), TRPC 1 (group 2), TRPC 3/6/7 (group 3), and TRPC 2 (group 4) [8], [9]. TRPCs operate as receptor-operated Ca^2+^ entry (ROCE) channels which are activated by agonist of receptors or SOCE channels which are activated by emptying of Ca^2+^ stores. All five TRPC proteins (TRPC1, 3, 4, 5, 6) detected were more highly expressed in the embryonic CNS compared with the adult, suggesting TRPC channels as candidates for mediating Ca^2+^ entry during proliferation of neuroepithelial cells [10]. TRPC as non-VOCCs is well established and involves in neural proliferation [6] and differentiation [11], [12]. Pla et al. showed that TRPC1 contributes to bFGF/FGFR-1-induced Ca^2+^ influx, which is involved in self-renewal of embryonic rat NSCs [6]. Moreover, TRPCs were reported to play a role in Netrin-1 or brain-derived neurotrophic factor (BDNF)-mediated growth cone turning, neuron survival and spine formation [11], [12]. Despite the key role of Ca^2+^ in development, little is known about the contribution of Ca^2+^ to cell-fate determination especially focused on the physiological function of TRPC as SOCE channels in the neural differentiation of NPCs.

In the present study, we identified and isolated NPCs with neural cell surface antigen of A2B5, used as a useful NPC marker [6], [13]–[16], by using magnetic activated cell sorting (MACS) from the cerebral tissues of the postnatal rat, and the majority of the isolated A2B5^+^ NPCs were able to differentiate into neural cells under the culture condition supplemented with fetal bovine serum (FBS), retinoic acid and brain-derived neurotrophic factor (BDNF). We asked whether TRPC as SOCE channel has influence on the neural cell fate decision of proliferating A2B5^+^ NPCs, and examined the role of TRPC in the differentiation of neural cells from A2B5^+^ NPCs. Here we found that the amplitude of SOCE is higher in cells differentiated from A2B5^+^ NPCs than in proliferating A2B5^+^ NPCs. Finally, pharmacological blockers of SOCE and siRNA against TRPC5 reduced the amplitude of SOCE and blocked the neural differentiation from A2B5^+^ NPCs.

## Materials and Methods

### Isolation of neuroglial progenitors

All animal study protocols were approved by the Seoul National University Hospital's Institutional Animal Care and Use Committee. Animal care was carried out in accordance with guidelines on the ethical use of animals approved by the Experimental Animals Committee of Seoul National University Hospital. All efforts were made to minimize the number of animals used and their suffering. Postnatal 12 day-old Spraque-Dawley (SD) rats (Koatech, Pyongtaek, Korea) were decapitated under ketamine anesthesia (50 mg/kg). To get a high yield of cell number, the whole cerebrum was lifted en bloc and washed extensively with phosphate-buffered saline (PBS). The cerebrum was mechanically dissected and enzymatically dissociated to single-cell suspensions using papain (Sigma, St. Louis, MO) and DNase I (Sigma) as previously described [14]. The cells were then suspended in DMEM/F12 (Invitrogen, Carlsbad, CA) supplemented with 5 µg/ml insulin (Sigma), 50 µg/ml transferrin (Sigma), 30 nM selenium chloride (Sigma), and 5 µg/ml fibronectin (Sigma) (ITSFn medium), followed by magnetic separation of A2B5^+^ cells. Magnetic separation was performed as previously described [13]. In brief, the cells were incubated with A2B5 supernatant (clone 105; American Type Culture Collection, Manassas, VA) for 30 minutes at 4°C. The cells were then washed three times with PBS containing 0.5% bovine serum albumin (BSA; Sigma) and 2 mM EDTA, and incubated with microbead-tagged mouse-specific rat IgM (1∶4; Miltenyi Biotech, Bergisch Gladbach, Germany) for 30 minutes at 4°C. The cells were washed, and separated using positive selection columns (Miltenyi Biotech).

### Fluorescence Activated Cell Sorting (FACS)

**Figure 1:**
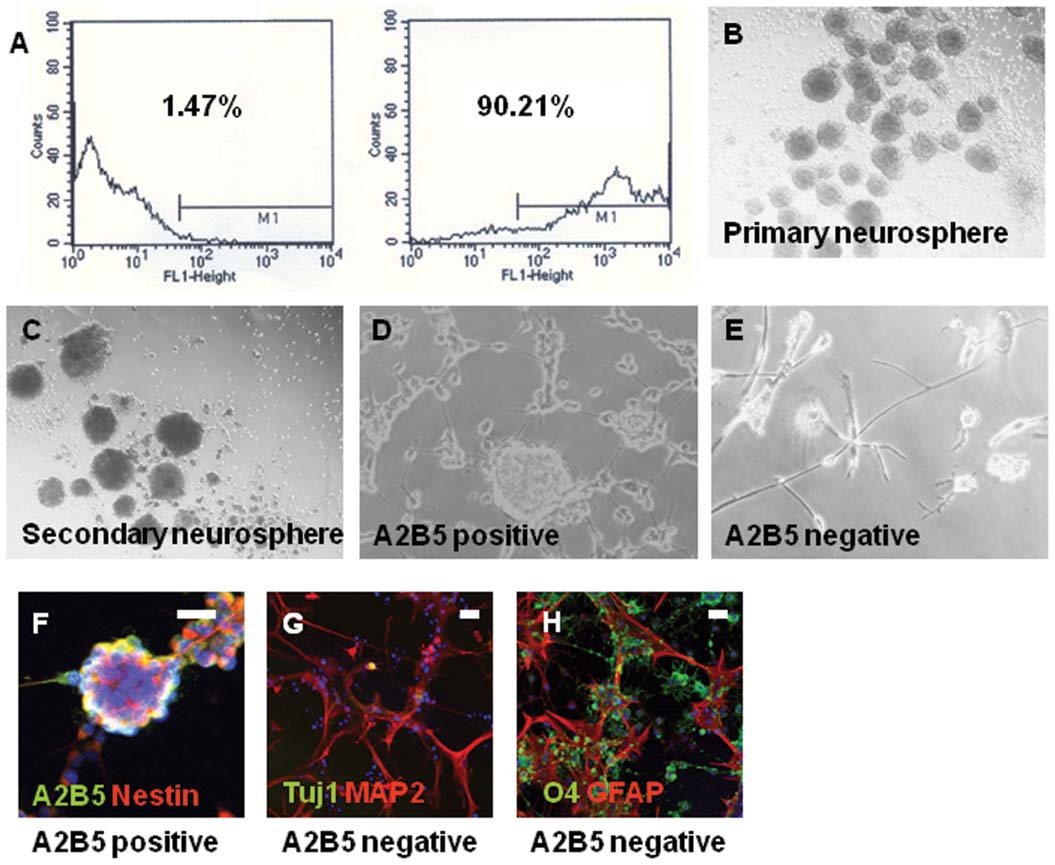
Isolation of A2B5^+^ cells and formation of neurospheres in the proliferation condition. (**A**)**:** FACS confirmed a high purity of A2B5^+^ MACS separation. A2B5^+^cell group after MACS separation contained 90.21% of A2B5^+^ cells (right). The 1.47% population is the same cells without secondary antibody. (**B**)**:** A2B5^+^ cells cultured in ITSFn formed numerous neurospheres at one week after**.** (**C**)**:** Secondary neurospheres were derived from cultured A2B5^+^ cells, after monthly passages. (**D**)**:** A2B5+ cells were plated onto fibronectin to record SOCE in proliferation medium, (**E**)**:** One or no sphere was found in cultured A2B5- cells until 4 weeks (**F**)**:** Neurospheres were A2B5^+^/Nestn^+^. (**G and H**)**:** Cultured A2B5- population consists of already-differentiated neuron, astrocyte, and oligodendrocyte. Scale bars, 50 µm.

The purity of isolated A2B5^+^ cells was evaluated by flow cytometry with a FACS Caliber machine (BD Biosciences, Franklin Lakes, NJ) at one week after primary culture (Figure 1A). The cells were incubated with A2B5 supernatant for 30 minutes at 4°C, and washed three times with PBS containing 0.5% BSA and 2 mM EDTA. Cells were then incubated with Alexa Fluor® 488-conjugated goat anti-mouse IgM (1∶400, Invitrogen) for 30 minutes on ice. Cell Quest acquisition and analysis software (BD Biosciences) was used to quantify the fluorescence signal intensities.

### Cell Culture in proliferation condition

Sort-purified A2B5^+^ NPCs were plated on 24-well plates at clonal cell densities of 1×10^6^ cells/ml. The cells were cultured under proliferation conditions in ITSFn medium supplemented with 20 ng/ml bFGF (Sigma), 2 ng/ml NT3 (R&D systems, Minneapolis, MN) and 20 ng/ml PDGF-AA (Sigma), a combination that was known to permit in vitro expansion of A2B5^+^ cells [13].

### Cell Culture in differentiation condition

At one week after isolation of A2B5^+^ cells, cells were switched to media for differentiation. At 40–60% confluence, the medium was replaced by neurotrophic medium (NM) that contains both serum and neurotrophic factors. NM consisted of ITSFn, supplemented with 0.5% fetal bovine serum, 0.5 mM retinoic acid (Sigma) and 20 ng/ml BDNF (Sigma). At 3 weeks after differentiation induction, the cells were processed for evaluation.

### Immunocytochemistry

The cells were rinsed with PBS, fixed with 4% paraformaldehyde for 30 minutes at room temperature, and washed three times with PBS. The cells subsequently permeabilized with PBS, 0.1% saponin and 1% normal goat serum (NGS), and blocked with PBS, 0.05% saponin and 5% NGS, each for 30 minutes. Primary antibodies including mouse anti-nestin (1∶400; Chemicon, Temecula, CA), mouse anti-Tuj1 (1∶400; Covance, Berkeley, CA), rabbit anti-GFAP (1∶900; Chemicon), mouse anti-O4 (1∶600; Chemicon), and calbindin D28k (1∶1000; Chemicon) were incubated overnight at 4°C. After rinsing, species- and isotype-specific goat secondary antibodies conjugated with Alexa Fluor® 568 or 488 (1∶400; Invitrogen) were applied for 1 hour at room temperature. Cells were mounted with antifading solution containing 4′-6-diamidino-2-phenylindole (DAPI; Vector Laboratories, Burlingame, CA), and observed under a confocal microscope (Zeiss, Oberkochen, Germany).

### Ca^2+^ imaging

Cells were plated onto poly-L-lysine coated 12 mm coverslips. Cells were loaded in 5 µM Fura 2-AM (Molecular Probes, Eugene, OR) and 0.01% pluronic acid in HEPES-buffered salt solution (HBSS) for 45 min at 37°C, and then unloaded in HBSS for another 15 min. Coverslips with Fura 2-AM loaded cells were then transferred to a perfusion chamber on the stage of an upright microscope (Olympus BX50). Cells were illuminated by a Xenon lamp and observed with a 40X UV water-immersion objective lens (NA: 0.8, LUMPlan FL 40· W; Olympus, Tokyo, Japan). For Fura 2-AM excitation, the shutter and filter wheel (polychrome-IV; TILL-Photonics, Martinsried, Germany) were controlled by Axon Imaging Workbench (AIW) software 2.1 (Axon Instruments, Foster City, CA) to provide sequential illumination at two alternating wavelengths, 340 and 380 nm. Fluorescence of Fura 2-AM was detected at an emission wavelength of 510 nm. Video images were acquired using an intensified CCD camera (LUCA; Andor, UK). Fluorescence emission ratios following excitation at 340 and 380 nm were calculated by dividing averaged pixel values in circumscribed regions of individual responding cells in the field of view. The values were exported from AIW to Origin 8.0 for additional analysis and plotting. The composition of the HBSS was (in mM); NaCl, 137; KCl, 5; MgSO_4_, 0.9; CaCl_2_, 1.4; NaHCO_3_, 3; Na_2_HPO_4_, 3; Na_2_HPO_4_, 0.6; KH_2_PO_4_, 0.4; glucose, 5.6; and HEPES, 20; pH 7.4. For the depolarization conditioning, Ca^2+^ imaging were performed in HBSS containing high K^+^ (133 mM K^+^ with substitution of Na^+^).

### Reverse transcription –PCR

Total RNA was isolated from sort-purified A2B5^+^ NPCs which had been cultured with either ITSFn medium supplemented with 20 ng/ml bFGF (Sigma), 2 ng/ml NT3 (R&D systems, Minneapolis, MN) and 20 ng/ml PDGF-AA (Sigma) or serum-containing NM for 3 weeks using RNeasy mini kit (Qiagen, GmbH, Germany). To eliminate genomic DNA contamination, DNase I digestion was performed using the RNase-free DNase set (Quiagen). First-strand cDNA synthesis was carried out by random priming of the total RNA using a random primer mixture (Invitrogen) and reverse transcriptase with superscript 3 (Invitrogen). PCR was performed to detect the different TRPC channel transcripts. PCR primer pairs are reported in Table S1. All designed primers were screened using BLAST (Basic Local Alignment Search Tool) to ensure specificity of binding. Primers were used at a concentration of 250 nM. Primers (2 µl of each) and template cDNA (2 µl) were added to the mixture. PCRs were performed by DNA thermal cycler (Applied Biosystems, CA), using Platinum PCR Supermix High Fidelity (Invitrogen). The PCR program was as follows: 10 min 94°C pre-run, 30 s at 94°C, 30 s at 55°C, 2 min at 72°C for 35 cycles, and 10 min 72°C post-run. No products were amplified in water.

### Treatment of 2-APB

The proliferation assay was done to show effects of SOCE inhibitor on the fate determination and survival of A2B5^+^ NPCs in the differentiation condition. Sort-purified A2B5^+^ NPCs were plated on 24-well plates at clonal cell densities of 1×10^6^ cells/ml, and cultured with ITSFn or NM media supplemented with 10, 50 and 100 µM 2-APB for five days, and then cells were switched to media without 2-APB. After 3 weeks, the cells were used for calcium imaging and cell proliferation assay.

### Cell proliferation assay

The proliferation assay was done to show effects of SOCE inhibitor on the fate determination and survival of A2B5^+^ NPCs in the differentiation condition. Cells were plated in triplicate onto 96-well plates with 2,000 cells/well along with appropriate controls containing only media and 2-APB. Cells were treated with 10, 50 and 100 µM 2-APB 24 h after plating. Cell growth was measured using the Cell Counting Kit-8 (CCK-8) following the manufacturer's (Dojindo Molecular Technologies, Inc., Gaithersburg, MD) protocol. 10 µL of CCK-8 reagent was added to each well and allowed to incubate for 3 h. The amount of CCK-8 reagent reduced to formazan by cellular dehydrogenase indicating cell viability was assayed by reading the absorbance at 405 nm on a 96-well plate reader. The absorbance reading was subtracted from the background control. The absorbance reading was averaged with three times measurement.

### Real time RT-PCR analysis

For real time PCR, total RNA was extracted using RNeasy mini kit (Qiagen). All primers and probes (Table 1) were purchased from Applied Biosystems (Lincoln, CA). For the SDS 7000 system reactions, a master mix of the following component was prepared at the indicated end concentration with 2.5 µl water, 2.5 µl forward primer (9 µM) and reverse primer (9 µM), 2.5 µl probe (2.5 µM), 12.5 µl TaqMan PCR 2x master mixture (Applied biosystems). Reverse transcribed total RNA (75 ng) in 5 µl was added as PCR template.

**Table 1:** Differential expression of TRPC subtype between differentiated cells and A2B5 ^+^ NPCs.

Subtypes	Differential expression ratio of mRNA	TaqMan assay
TRPC1	<0.1	Rn00585625_m1
TRPC2	1.5	Rn00575304_m1
TRPC3	6.9	Rn005729285_m1
TRPC4	0.1	Rn00584853_m1
TRPC5	42.5	Rn00590142_m1
TRPC6	30.6	Rn00677599_m1
TRPC7	–	Rn01448763_m1

Abbreviations:TRPC, canonical transient receptor potential channel; NPCs, neural progenitor cells.

Relative quantitative real-time PCR on 96-well optical plates was performed by using the above reagents and analyzed on an ABI Prism 7000 Sequence Detection System (Perkin-Elmer Applied Biosystems, Lincoln, CA). The following PCR conditions were used: After initial activation of uracyl-N-glycosylase at 50°C for 2 min, AmpliTaq Gold was activated at 95°C for 10 min. The subsequent PCR condition consisted of 45 cycles of denaturation at 95°C for 15 s and annealing extension at 60°C for 1 min per cycle. During the PCR amplification, the amplified products were measured continuously by determination of the fluorescence emission. The expression level of target gene was normalized to internal Rat GAPDH and represented as relative expression. To confirm a constant housekeeping gene expression level in the investigated total RNA extractions, a GAPDH real time PCR was performed. Real time PCR was qualified in the SDS 7000 (Applied Biosystems) with the Rat GAPD (GAPDH) Endogenous Control (VIC / MGB Probe, Primer Limited).

### siRNA and Transfection

Cells were grown up to 50% confluence and were incubated in serum-free medium for 72 h with the siRNA (50 pmol) in the presence of Lipofectamine 2000 (Invitrogen Life Technology, Carlsbad, CA) as described by the manufacturer. The sequences used are reported in Table S2. To assess transfection efficiency, we used the BLOCK-iT™ Alexa Fluor® Red Fluorescent Oligo that is not homologous to any known genes as transfection efficiency detector and a negative control to ensure against induction of nonspecific cellular events caused by introduction of the Oligo into cells.

### Single cell–PCR analysis

After Ca^2+^ imaging recoding, the single cell was isolated with 10 µl of DEPC-water. PCR was performed to detect the different TRPC channel transcripts, MAP2, Nestin. PCR primer pairs are reported in Table S1. For the first of PCR reaction, 17.5 µl of nuclease free H_2_O, 2.5 µl of AccuPrime 10X PCR buffer (Invitrogen) containing deoxynucleoside triphosphate mix (2.5 mM), and 1 µl of AccuPrime Tag (Invitrogen) were mixed. Primers (1 µl of each) and distilled templates of single cell (2 µl) were added to the mixture. The PCR program was as follows: 5 min 94°C pre-run, 30 s at 94°C, 30 s at 55°C, 1 min at 72°C for 30 cycles, and 10 min 72°C post-run. For the second of PCR reaction, 9.5 µl of nuclease free H_2_O, 2.5 µl of AccuPrime 10X PCR buffer (Invitrogen) containing deoxynucleoside triphosphate mix (2.5 mM), and 1 µl of AccuPrime Tag (Invitrogen) were mixed. Primers (1 µl of each) and products of the first PCR (10 µl) were added to the mixture. The PCR program was the same as for the first PCR. No products were amplified in water.

### Statistical analysis

Statistical analysis was performed using Origin 8.0. Data are expressed as mean values ± standard error of mean (SEM). Statistical analyses were performed by using Student T-test. The differences between groups were considered significant at p<0.05.

## Results

### Isolation of A2B5^+^ NPCs and Characterization

**Figure 2:**
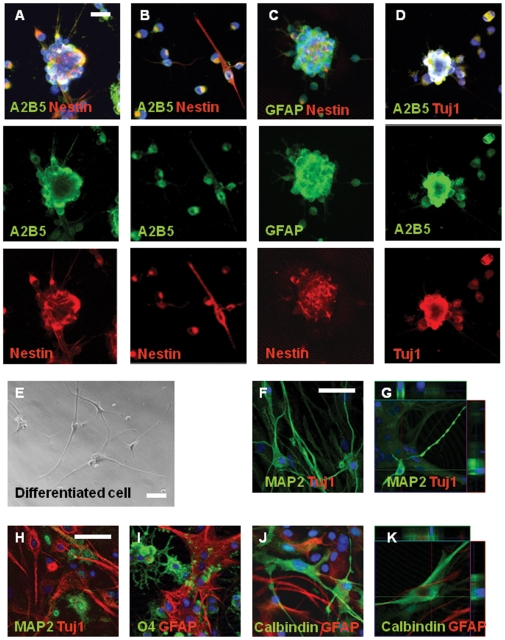
Identification of neural stem cell characteristics of A2B5^+^ NPCs and differentiation of A2B5^+^ NPCs into mature neuronal and glial cells. Many cells in the neurospheres stem cell-like characteristics. (**A,C,and D**): Neurosphere was A2B5^+^/Nestn^+^, GFAP^+^/Nestin^+^, and A2B5^+^/Tuj1^+^ confirming their stem cell-like characteristics. Although there is a difference in intensity, each single channel describes co-expression (**B**): Two percent of A2B5^+^ NPCs adhered to plates and were A2B5^+^/Nestn^+^. (**E**): Many cells in these cultures manifested progressive neuronal morphological maturation during 3 weeks in differentiation condition. (**F-I**): A2B5^+^ NPCs cultured in differentiation media developed cellular processes and differentiated into neuronal cells (MAP2^+^/Tuj1^+^), astrocytes (GFAP^+^) and oligodendrocytes (O4^+^). (**J and K**): The differentiated neuronal cells were also positive for calbindin D28k, intracellular calcium-binding proteins of the EF-hand related to calmodulin and troponin-C. Scale bars, 50 µm.

The number of the A2B5^+^ NPCs isolated from rat cerebrum was about 2X10^6^ per rat brain after MACS sorting. Whole cerebrums were used to get a high yield of cell number. The purity of isolated A2B5^+^ NPCs was evaluated by flow cytometry at one week after primary culture, and 90.21% of the sorted cells were A2B5^+^ (Figure 1A). A2B5^+^ NPCs are non-adherent cells, and proliferated actively in ITSFn, a stem cell-enrichment medium, whereas two percent of A2B5^+^ NPCs adhered to plates. We used fibronectin to attach cells on cover slip to record SOCE in proliferation medium (Figure 1D). Immunofluorescence was performed on the A2B5+ NPCs after MACS sorting; A2B5^+^ NPCs expressed both A2B5 and Nestin, a neural stem cell marker (Figure 1F). No mature neuron, astrocyte, or oligodendrocyte was found. Therefore, the possibility that some of the A2B5+ population consist of already-differentiated oligodendrocytes is very low. Neurospheres were formed 4 to 5 days after MACS, and the neurospheres were maintained for more than 4 weeks (Figure 1B). Secondary neurospheres were derived from dissociated cells, after monthly passages (Figure 1C), and multipotent capacity was confirmed. We used fibronectin to attach cells on cover slip to record SOCE in proliferation medium (Figure 1D). Immunocytochemistry was performed at 1 week after MACS, and most of the neurospheres were positive for A2B5 and the majority of them co-expressed Nestin (Figure 2A), GFAP, a glial cell or a subventricular zone stem cell marker (Figure 2C); Tuj1, an early neural cell marker (Figure 2D). Tuj1 expression indicates that the neuropheres are committed to neuronal lineage. Although there is a difference in intensity, each single channel describes co-expression (Figure 2A-D). Two percent of A2B5^+^ NPCs adhered to plates and were A2B5^+^/Nestn (Figure 2B). We cultured A2B5^-^ population in identical conditions, however, one or no sphere was found until 4 weeks (Figure 1E). In addition, most of them were positive for MAP2, mature neural cell marker (Figure 1F); O4, oligodendrocyte marker (Figure 1H); or GFAP, astrocyte marker (Figure 1H).

### Induction of neuronal differentiation

**Figure 3:**
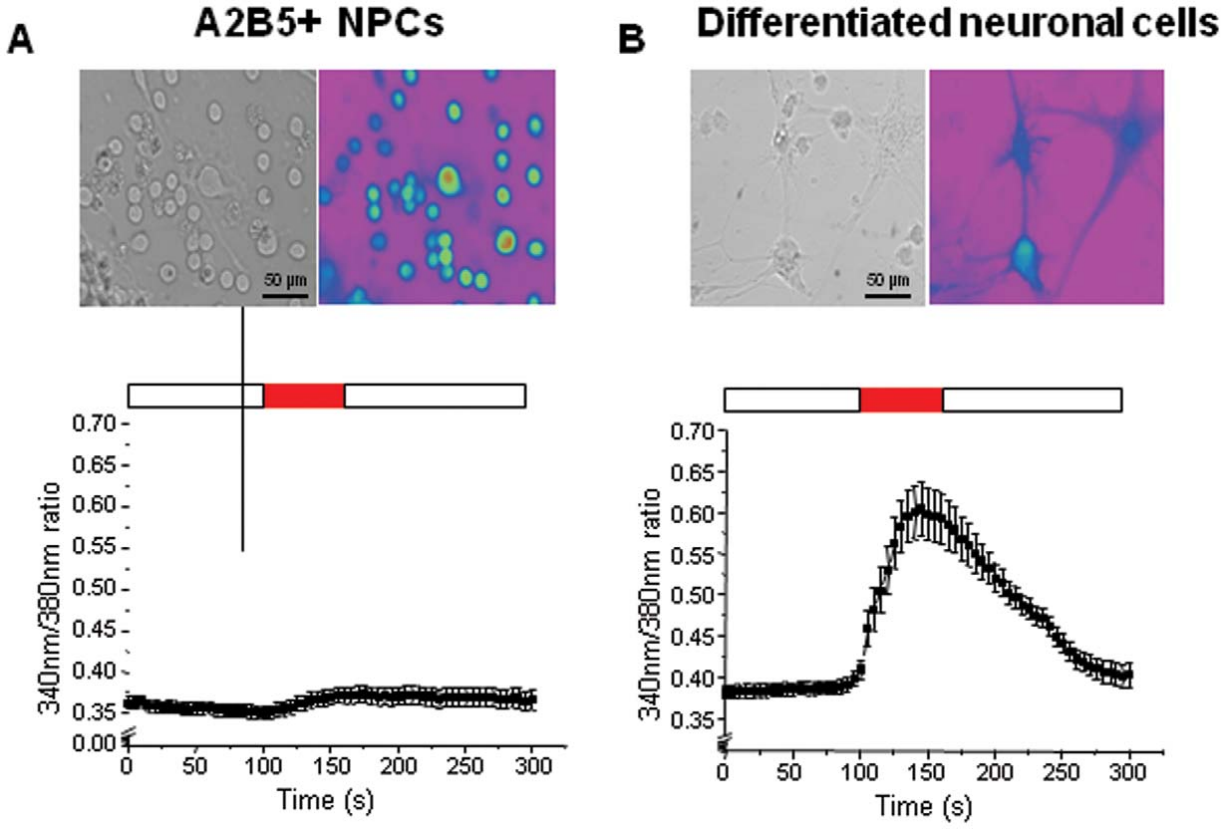
High KCl-induced Ca^2+^ transient in A2B5^+^+ NPCs and differentiated neuronal cells. (**A, B**)**:** Cells were pre-loaded with Fura 2-AM, washed and pre-incubated for at least 10 min prior to the addition of KCl (133 mM) for 60 sec. Upper panel show false-colour images of the cells illuminated at 340 nm. Lower trace shows high KCl-induced Ca2+ transient, in A2B5+NPCs (**A**) and differentiated neuronal cells (**B**) respectively. The time of KCl addition is indicated by the red. Error bars represent SEM. The data are representative of at least 3 separate experiments.

A2B5^+^ NPCs can generate passageable neurospheres that give rise to neuronal cells, astrocytes and oligodendrocytes. When switched from ITSFn to media for differentiation, A2B5^+^ cells demonstrated morphological changes after 3 or 4 days. Floating cells adhered to plates and developed cellular processes (Figure 2E). Many cells in these cultures manifested progressive neuronal morphological maturation during 3 weeks *in vitro*. Immunocytochemistry was performed at 3 weeks after the change from ITSFn to media for differentiation demonstrated that the cells were differentiated into neuronal cells (MAP2^+^/Tuj1^+^) (Figure 2F-H), astrocytes (GFAP^+^) (Figure 2I) and oligodendrocytes (O4^+^) (Figure 2I). The majority of neuronal cells were MAP2^+^/Tuj1^-^ (Figure 2F and G), but some of them were MAP2^+^/Tuj1^+^ (Figure 2H). The differentiated neuronal cells were also positive for calbindin D28k, intracellular calcium-binding proteins of the EF-hand related to calmodulin and troponin-C (Figure 2J and K). To test the neuronal nature of the differentiated cells from A2B5^+^ NPCs, we examined the changes in intracellular free Ca^2+^ concentration ([Ca^2+^]_i_) in response to depolarization of membrane potential with superfusion of high KCl (133 mM). We performed on dual-wavelength excitation microfluorimetry using in fura-2-loaded cells. High KCl (133 mM) bathing solution for 60 sec increased [Ca^2+^]_i_ in differentiated neuronal cells (Figure 3). In contrast, cells in proliferation media had no changes in [Ca^2+^]_i_ by membrane depolarization with high KCl.

### SOCE is higher in differentiated neuronal cells than proliferating A2B5^+^ NPCs

**Figure 4:**
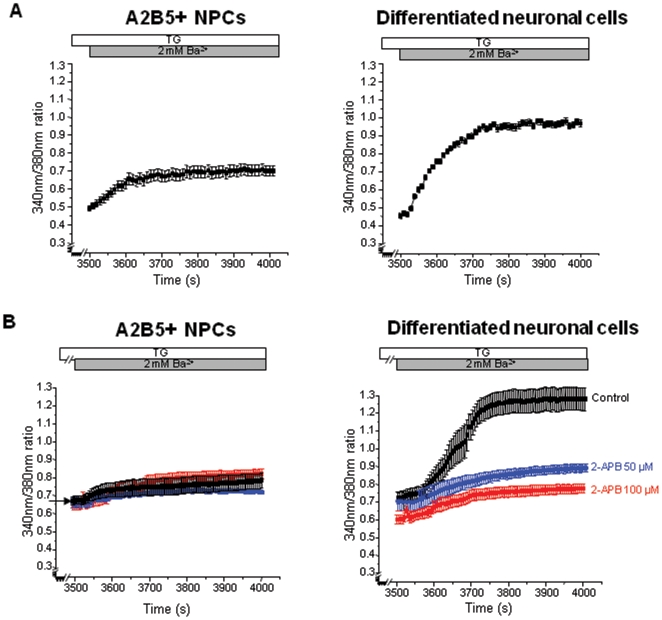
TG-stimulated Ca^2+^-influx measured in A2B5^+^ NPCs *versus* differentiated neuronal cells. (**A**)**:** In the absence of calcium, Ca^2+^ stores were released with 1 µM thapsigargin and then 2 mM Ba^2+^ added. TG-stimulated Ba^2+^ influx was monitored in A2B5^+^ NPCs and differentiated neuronal cells. (**B**)**:** TG-stimulated Ba^2+^ influx was measured for the control (0 µM) or the experimental groups for which 2-APB (50 or 100 µM) was added into the camber. After store depletion, TG-stimulated Ba^2+^ influx was inhibited by the addition of 2-APB. Each trace is the average of A2B5^+^ NPCs or differentiated neuronal cells. The time of Ba^2+^ addition is indicated by the dark grey and that of 2-APB is done by the grey. Error bars represent SEM. The data are representative of at least 3 separate experiments.

We recorded SOCE of cells under proliferation and differentiation conditions. SOCE were monitored by thapsigargin (TG)-stimulated Ba^2+^ influx. Cells were superfused in HBSS, then shifted to a Ca^2+^-free HBSS and TG was added at a dose of 1 µM. TG depleted intracellular Ca^2+^ store by inhibiting SERCA pump-mediated Ca^2+^ reuptake into the Ca^2+^ store. We chose Ba^2+^ as a charge carrier because Ba^2+^ is permeable through Ca^2+^ entry pathway but not pumped by the Ca^2+^-ATPases [19], [20]. After TG-stimulation (3500s), 2 mM Ba^2+^ was added to the bathing solution and Ba^2+^ entry was monitored. TG-stimulated Ba^2+^ influx was significantly higher in differentiated neuronal cells (Figure 4A; n = 161, 0.51±0.01) than in proliferating A2B5^+^ NPCs (Figure 4A; n = 94, 0.2±0.02, p<0.01 compared with differentiated cells). Higher SOCE in differentiated neuronal cells could result from hyperpolarization of membrane potential and higher driving force for Ba^2+^ across the membrane. To exclude this possibility, we eliminated membrane potential using high potassium bathing solution (133 mM KCl). Even in this condition, TG-stimulated Ba^2+^ influx was still significantly higher in the differentiated neuronal cells than in the proliferating A2B5^+^ NPCs (Figure S1). These data indicated that the amplitudes of SOCE were higher in the differentiated neuronal cells than the A2B5^+^ NPCs.

We evaluated the effects of 2-APB, a known inhibitor of SOCE, on TG-stimulated Ba^2+^ influx (Figure 4B). Addition of 2-APB in the bathing solution induced inhibitions of TG-stimulated Ba^2+^ influx in the differentiated neuronal cells (50 µM: 0.13±0.03, n = 46, P<0.05; 100 µM: 0.13±0.03, n = 48, P<0.05). On the contrary, 2-APB did not change TG-stimulated Ba^2+^ influx in the proliferating A2B5^+^ NPCs. Application of other inhibitor of SOCE, ruthenium red (RR), significantly inhibited TG-stimulated Ba^2+^ influx in the differentiated neuronal cells (Figure S2; 20 µM: 0.18±0.01, n = 40, P<0.05).

### SOCE Inhibitor during induction of differentiation decreases SOCE

**Figure 5:**
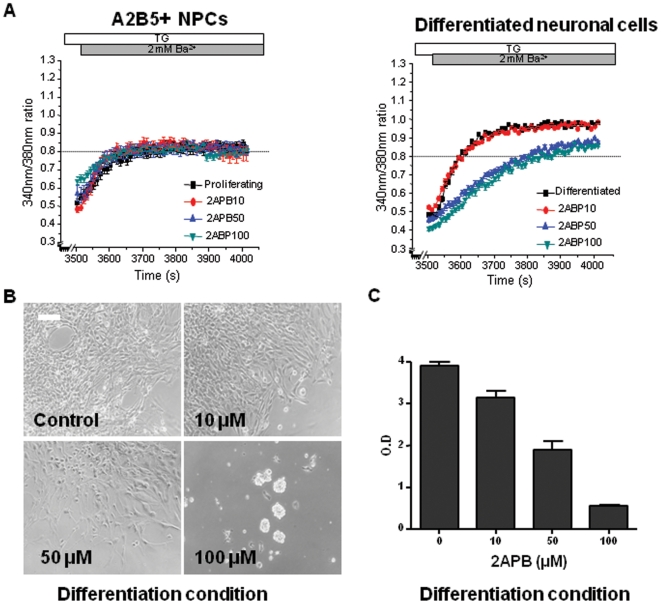
Effect of 2-APB on TG-stimulated Ca^2+^-influx and cell viability in differentiated neuronal cells. The A2B5^+^ NPCs were cultured in NM media supplemented with 10, 50 and 100 µM 2-APB for five days, and then the cells were switched to media without 2-APB. (**A**)**:** After 3 weeks, TG-stimulated Ba^2+^ entry was monitored in A2B5^+^ NPCs and differentiated neuronal cells. After store depletion, TG-stimulated Ba^2+^ influx was inhibited by the addition of 2-APB in differentiated media. Each trace is the average of A2B5^+^ NPCs or differentiated neuronal cells. The time of Ba^2+^ addition is indicated by the dark grey and that of 2-APB is done by the grey. Error bars represent SE. The data are representative of at least 3 separate experiments. (**B**)**:** After 3 weeks, the A2B5^+^ NPCs treated with 10 µM 2-APB normally differentiated into mature neuronal cells. However, the rate of differentiation of the cells significantly reduced in the media supplemented with 50 µM 2-APB, and the cells hardly differentiated into mature neuronal cells in the media supplemented with 100 µM 2-APB. (**C**)**:** The proliferation assay was done to show effects of SOCE inhibitor on the fate determination and survival of A2B5^+^ NPCs in the differentiation condition. For proliferation assay, the A2B5^+^ NPCs were treated with 2-APB at the indicated concentrations (10, 50 and 100 µM) 24 h after plating. Data was expressed as the mean optical density of 3 readings. Error bars indicate the standard deviation. Scale bars, 50 µm.

To evaluate the effects of SOCE inhibitor on the fate determination of A2B5^+^ NPCs, A2B5^+^ NPCs were cultured in ITSFn or NM media supplemented with 10, 50 and 100 µM 2-APB for 5 days, and then cells were switched to media without 2-APB up to 3 weeks. The A2B5^+^ NPCs were differentiated into mature neuronal cells in the differentiation media supplemented with 10 µM 2-APB (Figure 5B). On the other hand, the neuronal differentiation of the cells significantly decreased in the media supplemented with 50 µM 2-APB, and the cells were hardly differentiated into mature neuronal cells in the media supplemented with 100 µM 2-APB. The amplitudes of TG-stimulated Ba^2+^ influx were significantly inhibited by 10, 50 and 100 µM 2-APB in the culture of differentiation media (Figure 5A). Furthermore, inhibition of SOCE reduced the cell viability dose-dependently during differentiation culture conditions with application of several doses of 2-APB (Figure 5C). The proliferation assay was done to show effects of SOCE inhibitor on the fate determination and survival of A2B5^+^ NPCs in the differentiation condition. As shown in Figure 5C, treatment of 2-APB reduced cell viability, proving the significant role of SOCE in the differentiation of A2B5^+^ NPCs. After 3 weeks, the A2B5^+^ NPCs treated with 10 µM 2-APB normally differentiated into mature neuronal cells. However, the rate of differentiation of the cells significantly reduced in the media supplemented with 50 µM 2-APB, and the cells hardly differentiated into mature neuronal cells in the media supplemented with 100 µM 2-APB.

Taken together, these data showed that the amplitudes of SOCE were elevated in the differentiated neuronal cells under the differentiation culture conditions, and the SOCE inhibitor might block the differentiation of A2B5^+^ NPCs causing cell death.

### Differential expression of TRPC subtypes between differentiated neuronal cells and A2B5 ^+^ NPCs

The TRPC family has been reported to be expressed in many kinds of stem cells. A2B5^+^ NPCs also expressed TRPC1 to 7, but TRPC2, a known pseudo gene (Figure S3). To quantitatively compare differential expression of TRPC between proliferating A2B5^+^ NPCs and differentiated neuronal cells, we performed real time RT-PCR analysis. Table 1 demonstrates that the gene expression of TRPC1 and TRPC4 were down-regulated in the differentiated neuronal cells compared to A2B5^+^ NPCs. Meanwhile, the gene expression of TRPC3, TRPC5, and TRPC6 was up-regulated in the differentiated neuronal cells compared to A2B5^+^ NPCs. Significantly, the expression level of TRPC5 and TRPC6 was up-regulated 42.5 folds and 30.6 folds in differentiated neuronal cells, respectively. Taken together, this result implies that TRPC5 and TRPC6 are highly expressed in differentiated neuronal cells compared with A2B5^+^ NPCs.

### Knockdown of TRPC5 attenuates SOCE and neural differentiation of the A2B5^+^ NPCs in the differentiation conditions

**Figure 6:**
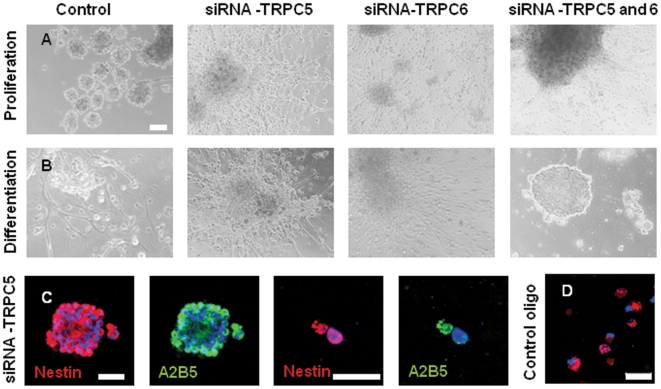
Effect of siRNA targeting TRPC5 and TRPC6 on neuronal differentiation of NPCs in the differentiation conditions. (**A, B**)**:** A2B5^+^ NPCs were transfected with siRNA against TRPC5, TRPC6, and both of them, respectively, and cultured in both proliferation and differentiation condition for 3 weeks. The proliferation rate in the treated cells was lower than control, and there was no significant difference in morphology or immunophenotype. (**C**): Confocal microscopic image shows that most cells treated with siRNA of TRPC5 and TRPC6 were Nestin^+^/A2B5^+^ in the differentiation condition. (**D**): To assess transfection efficiency, the BLOCK-iT™ Alexa Fluor® Red Fluorescent Oligo was used. Although complete knockdown is difficult, prevalent red fluorescence proved high transfection efficiency. Scale bars, 50 µm.

**Figure 7:**
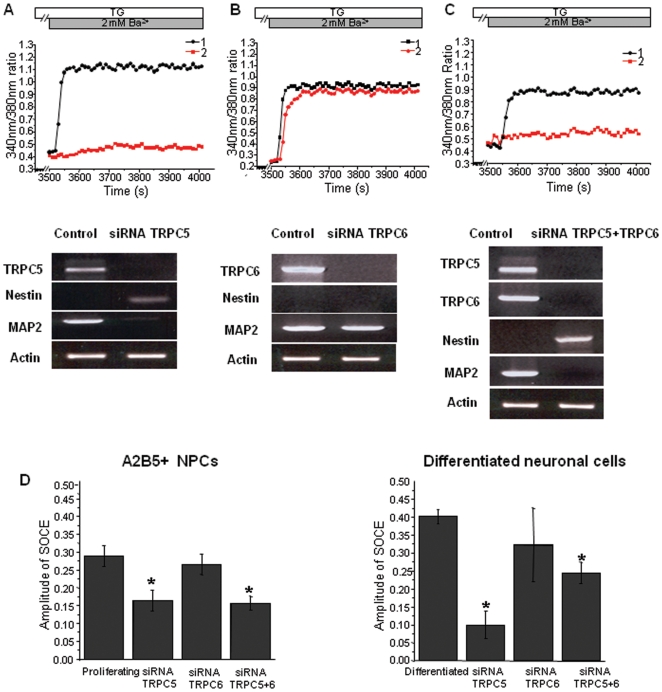
Effect of expressing siRNA targeting TRPC5 and TRPC6 on TG-stimulated Ca^2+^-influx in the neuronal differentiation. A2B5^+^ NPCs were transfected with siRNA of TRPC5, TRPC6, and both of them, respectively and cultured in the differentiation condition for 3 weeks. (**A-C**)**:** TG-stimulated Ba^2+^ entry was monitored in control cells and cells transfected with siRNA of TRPC5 (A) or TRPC6 (B) or both of them (C). TG-stimulated Ba^2+^ influx was inhibited by siRNA of TRPC5 as well as siRNA of both TRPC5 and TRPC6. After recording, we performed single cell PCR in neuronal differentiation (black; siRNA-untransfected cell, red; siRNA-transfected cell). Cell transfected with siRNA of TRPC5 was Nestin^+^/MAP2^-^, although that transfected siRNA of TRPC6 was Nestin^-^/MAP2^+^. (**D**)**:** For average data, A2B5^+^ NPCs were transfected with siRNA and cultured in the differentiation condition. TG-stimulated Ba^2+^ influx was significantly inhibited by siRNA TRPC5 and TRPC5+TRPC6. The time of Ba^2+^ addition is indicated by the dark grey. Error bars represent SEM. The data are representative of at least 3 separate experiments. *, P<0.01 compared with corresponding control without siRNA TRPC.

To further investigate whether TRPC5 and TRPC6 mediate SOCE, the A2B5^+^ NPCs were individually transfected with siRNA of TRPC5, TRPC6, or both of them, and then cultured in proliferation and differentiation conditions for 3 weeks. There was no significant effect of siRNA on morphology or immunophenotype in the proliferation condition (Figure 6A). On the other hand, down-regulation of either TRPC5 or TRPC6 or both of them using siRNA significantly suppressed neuronal differentiation of the A2B5^+^ NPCs in the differentiation condition (Figure 6B). Furthermore, the cells treated with siRNA of TRPC5 and TRPC6 were Nestin^+^/A2B5^+^, showing that they didn't differentiate into mature cells in the differentiation medium (Figure 6C). This result suggests that TRPC5 and TRPC6 as SOCE channel play a role in the induction of neural differentiation from A2B5^+^NPCs. When A2B5^+^ NPCs were treated with siRNA of both TRPC5 and TRPC6, the cells hardly proliferated and differentiated in the differentiation condition (Figure 6B). High efficiency of transfection was confirmed by the BLOCK-iT™ Alexa Fluor® Red Fluorescent Oligo, that is not homologous to any known genes as transfection efficiency detector and a negative control to ensure against induction of nonspecific cellular events caused by introduction of the Oligo into cells (Figure 6D). Although complete knockdown is difficult, prevalent red fluorescence proved high transfection efficiency. Moreover, single cell RT-PCR proved knockdown of TRPC5 and TRPC6 (Figure 7A -C).

In the differentiation condition, the cell treated with siRNA of TRPC5 showed decrease in TG-stimulated Ba^2+^ influx and did not express MAP2, but expressed Nestin (Figure 7A). The cell treated with both siRNAs of TRPC5 and TRPC6 also showed decrease in TG-stimulated Ba^2+^ influx and did not express MAP2, but expressed Nestin (Figure 7C). However, TG-stimulated Ba^2+^ influx was not inhibited in the cell treated with siRNA of TRPC6. This cell did not express Nestin, but expressed MAP2 (Figure 7B). Significantly, knock-down of TRPC5 resulted in inhibition of TG-stimulated Ba^2+^ influx in cell in the differentiation media (Fig. 7D, 0.10±0.04, n = 42, P<0.01). In contrast to TRPC5, knock-down of TRPC6 had no significant effect on the amplitude of TG-stimulated Ba^2+^ influx in cell in the differentiation media (Figure 7D). Additionally, TG-stimulated Ba^2+^ influx was also inhibited in the cells co-transfected with both siRNAs of TRPC5 and TRPC6 upon differentiation conditions, and the extent of inhibition of TG-stimulated Ba^2+^ influx was slightly decreased compared with the result of the TRPC5 knock-down (Figure 7D). Taken together, these results suggest that not TRPC6 but TRPC5 as SOCE channel plays a major role in the induction of neuronal differentiation from A2B5^+^NPCs.

## Discussion

In the previous study, all five TRPC proteins (TRPC1, 3, 4, 5, 6) detected were more highly expressed in the embryonic CNS compared with the adult, suggesting TRPC channels as candidates for mediating Ca^2+^ entry during proliferation of neuroepithelial cells [10]. In this study, we demonstrate a significant role of TRPC 5 in the middle development stage between embryonic and adult stages of brain development, not elucidated in the previous studies. We used A2B5^+^ magnetic sorting to isolate and purify the neural progenitor cells from the postnatal-12-day rat cerebrum, falling between the embryonic and adult stages of brain development.

The cell surface antigen A2B5 recognizes a sialoganglioside/ sulfatide epitope on various neural and glial subtypes [6], [13]–[18], and it is also present on proliferative human embryonic stem cell-derived neural cells as well as on cells of the glial lineage [16]. It has been reported that A2B5-defined white matter progenitor cells (WMPCs) yield neurospheres, and these spheres generate all major neural phenotypes as well as glia *in vivo* and *in vitro* [13]. Pla et al. utilized the sort-purified A2B5^+^ cells as proliferating neural stem cells harvested from embryonic rat telencephalon in their study of the role of TRPC1 in basic fibroblast growth factor (bFGF)/FGF receptor-1-induced Ca^2+^ entry [6]. Maric et al. also utilized the expression of A2B5 and JONES (anti-9-O-acetylated GD3) protein as the positive marker of neuroglial progenitor cells in multiepitope labeling of E13 rat telenchephalon to investigate dynamically changing anatomical distributions of neural progenitors at the beginning of neurogenesis [19].

In this study, A2B5+ NPCs are non-adherent cells in the proliferation stage without fibronectin coating, and the neurospheres were formed 4 to 5 days after primary culture (Figure 1B). Immunofluorescence was performed on the A2B5+ NPCs after MACS sorting, and found no mature neuron, astrocyte, and oligodendrocytes. Therefore, the possibility that some of the A2B5+ NPCs consist of already-differentiated oligodendrocytes is very low. Stem cell marker expression of A2B5+ NPCs-derived neurospheres was demonstrated by immunofluorescence. Most of A2B5+ NPCs-derived neurospheres expressed A2B5, and the majority of them co-expressed Nestin (Figure 2A), GFAP, a glial cell or a subventricular zone stem cell marker (Figure 2C); Tuj1, an early neural cell marker (Figure 2D). Tuj1 expression indicates that the neuropheres are committed to neuronal lineage. Self-renewal and multipotency were also confirmed. Secondary neurospheres were derived from cultured A2B5^+^ cells, after monthly passages (Figure 1C), and multipotent capacity was confirmed. Neurpsphere-derived cells differentiated to neuron, astrocyte, and oligodendrocyte, indicating multipotency. A2B5^+^ NPCs cultured in differentiation media developed cellular processes and differentiated into neuronal cells (MAP2^+^/Tuj1^+^), astrocytes (GFAP^+^) and oligodendrocytes (O4^+^) (Figure 2F-I). The differentiated neuronal cells were also positive for calbindin D28k, intracellular calcium-binding proteins of the EF-hand related to calmodulin and troponin-C (Figure 2J and K). Moreover, Ca2+ response by high K+ proved functionally mature neuron activity in neuronal cells, differentiated from A2B5^+^ NPCs (Figure 3B). On the other hand, one or no sphere was found until 4 weeks in A2B5- cells in identical conditions (Figure 1E), and the cultured A2B5- cells consists of already-differentiated neuron (Figure 1F and H).

Recently the role of TRPC in the development of CNS was emphasized in many reports. TRPC is expressed in the embryonic CNS, suggesting important regulators during CNS development [10]. Several studies have reported that TRPC regulates neuronal development via a modulation of non-VOCCs. TRPC contributes to bFGF-dependent neural stem cell proliferation [6]. Moreover, TRPCs were reported to play a role in Netrin-1 or BDNF-mediated growth cone turning, neuron survival and spine formation [11], [12]. However, the physiological function of TRPC as SOCE in the differentiation of NPCs is still not studied. In this study, we focused on the physiological function of the TRPCs as SOCE in the differentiation of NPCs. In the line of previous reports, the most interesting finding of the present study is that the amplitudes of SOCE were increased in differentiated neuronal cells. In addition, the application of 2-APB as SOCE inhibitor reduced the amplitudes of SOCE in the differentiation condition (Figure 5A), and the neuronal differentiation of the cells significantly decreased in the media supplemented with 50 or 100 µM 2-APB in the differentiation condition (Figure 5B). This result implies a key role of SOCE in the neuronal differentiation. Consistent with our result, other group's findings show that SOCE is up-regulated during the differentiation of the H19-7 hippocampal cell line [20]. SOCE could be also developmentally regulated in many cell types [21], [22]. In this study, it is suggested that Ca2+ entry mediated by SOCE channel might play an important role in the determination of neuronal cell fate of NPCs.

Although, the age of the tissue studied can make a different result, and the extent of expression of TRPC is different according to developmental stage, there is a correlation between this study and previous studies. In this study, it is demonstrated that the neuronal differentiation of A2B5^+^ NPCs was closely associated with the change of the up-regulated expression of TRPC5 and 6 combined with down-regulated expression of TRPC1 as SOCE channel (Table 1). In addition, it was shown that not TRPC6 but TRPC5 as SOCE channel participates in the induction of neural differentiation from A2B5^+^ NPCs. Although, application of the SOCE inhibitor 2-APB had no effect in A2B5+ NPC (Figure 4), the knock-down of TRPC5 with siRNA significantly blocked the SOCE (Figure 7D). We speculate that the difference between two results can be ascribed to the fact that siRNA for TRPC5 is more specific than 2-APB. A2B5^+^ NPCs treated with siRNA of TRPC5 hardly proliferated in the proliferation condition, although the expression level of TRPC5 in A2B5^+^ NPCs was not high compared with differentiated cell. This result is consistent with the previous study, which shows that TRPC5 predominantly expresses in the CNS and is involved in proliferation of embryonic rat stem cells [23]. In addition, our finding of up-regulation and activation of TRPC5 in the neuronal cells at stage of differentiation parallels with the previous studies [24], [25]. Wu et al. showed that TRPC5 was required for differentiation. In this case, TRPC5 activation and subsequent neurite outgrowth were dependent phospholipas Cγ and PI3 kinase signal. The function of TRPC5 was to initiate the early stage of neurite sprouting [24]. In addition, Davare et al. demonstrated that TRPC5 activated Ca2+/Calmodulin Kinase Ιγ, which subsequently promoted axon formation [26]. Taken together, we suggest that the role of TRPC5 as SOCE channel is to regulate neuronal differentiation of A2B5^+^ NPCs. We postulate that expression of TRPC5 increases in the A2B5+ NPCs during the development stage. Subsequently, these activated TRPC5 may promote the neuronal differentiation, resulting in high expression of TRPC5 in the neuronal cells compared to the A2B5^+^ NPCs. The underlying mechanism: the downstream effects of SOCE on the transcriptional regulation of key genes involved in neurodevelopment in this system, needs to be further studied.

Unlike other TRPCs family, TRPC5 highly expresses in the plasma membrane and the perinuclear zone of hippocampal neuron as well as in the nuclei of neuron [24], [25]. These reports may suggest that TRPC5 can be a candidate molecule to influence the transcriptional regulation via changing of Ca^2+^ channel pathway and its possible connection between plasma membrane to nuclei. Pla et al. investigated the involvement of TRPC1 in bFGF-mediate Ca2+ entry and proliferation of embryonic rat neural stem cells [6]. They also reported that strong expression of TRPC-1 as well as TRPC2, 3, 4 and TRPC6 with barely expressed TRPC5 and TRPC7 as shown in the sort purified A2B5^+^ NPC harvested from postnatal-12-day rat cerebrum in this study (Figure S3). TRPC5 was expressed in the dissociated cells of the entire telencephalon and in the neural progenitor subpopulation but not in NSCs, which expressed TRPC1–4 and 6 [13]. However, they did not notice the change of over-expression of TRPC5 that was one of the main findings in the change of TRPC subtypes as well as marked decrease of TRPC1 during the neural differentiation from the A2B5^+^ NPCs. Furthermore, it was demonstrated in this study that siRNA of TRPC5 blocked the neuronal differentiation from A2B5^+^ NPCs and reduced the rise of SOCE. However, knock-down of TRPC6 had no significant effect on the amplitude of SOCE indicating that not TRPC 6 but TRPC5 contributes to SOCE. In this study, the cells hardly proliferated and differentiated in the differentiation condition by simultaneous knock-down of TRPC5 and TRPC6, but the extent of inhibition against TG-stimulated Ba^2+^ influx was slightly decreased compared with the single knock-down of TRPC5. This decreased extent of inhibition might be caused by competition between siRNA of TRPC5 and TRPC6 as well as dilution effect. In general, endogenously expressed TPRC proteins form heteromultimers composed of members from the same subgroup. TRPC5 and TRPC6 belong to different TPRC subgroups and are unlikely to co-assemble. TRPC channels from the different subgroups can form heteromers under specific circumstances. TRPC3 and TRPC6 can form a heteromeric channel complex with TRPC1, TRPC4 and TRPC5 in rat embryonic brain, but not in adult brain [10]. Otherwise the presence or absence of TRPC5 and TRPC6 in association with TRPC1 may help to explain bFGF-activated current changes during the neuronal differentiation from A2B5^+^ NPCs possibly via formation of different heteromeric TRPC complexes such as heteromeric TRPC (1+5) and TRPC (1+3+5) channels as described previously [10]. In this regard, it is likely that the different combinations of heteromeric formation of TRPC members of the TRPC family might mediate the main driving forces during the process of the neuronal differentiation of NPCs. Further studies are needed how these TRPC channels interact during the neuronal differentiation from NPCs.

Recent studies on the molecular mechanism of SOCE has been identified the ER Ca^2+^ sensors, stromal interaction molecule, (STIM) and the plasma-membrane channels, Orai 1-3 [27]–[32]. Orai1 is the most potent to reconstitute Ca2+ influx in most cells, and its depletion has the highest impact on SOCE [27], [28]. Thus, we examined the expression change of STIM1 and Orais 1–3 between proliferating A2B5^+^ NPCs and differentiated cells, and whether knockdown of STIM1 and Orai1 causes a change in the differentiation potency of A2B5^+^ NPCs in differentiation condition. To quantitatively compare differential expression of SOCE-related genes between proliferating A2B5^+^ NPCs and differentiated neuronal cells, we performed RT-PCR (Figure S3) and real time RT-PCR analysis (Table 1). The expression of TRPC5 and TRPC6 was up-regulated in the differentiated neuronal cells compared to A2B5^+^ NPCs (Table 1). The expression level of TRPC5 and TRPC6 was up-regulated 42.5 folds and 30.6 folds in differentiated neuronal cells, respectively. Meanwhile, the expression of STIM1 and Orai1-3 was not significantly up-regulated in the differentiated neuronal cells compared to A2B5^+^ NPCs (Table S3). The expression level of STIM1 and Orai1-3 was up-regulated 2.5, 1.8, 1.7, and 1.5 folds in differentiated cells, respectively. To further investigate whether STIM1 and Orai1 mediate cell differentiation, A2B5^+^ NPCs were individually transfected with siRNA of STIM1, Orai1, or both of them, and then cultured in differentiation conditions. As shown in Figure S4A, B, and C, most cells treated siRNA of STIM1, Orai1, or both of them manifested progressive morphological maturation during 2 weeks in differentiation condition, and the cells were differentiated into neuronal cells (MAP2^+^/Tuj1^+^), astrocytes (GFAP^+^). In additioin, RT-PCR showed that there was no significant effect of siRNA on MAP2 expression (). In contrast, the cells treated with siRNA of TRPC5 and TRPC6 were Nestin^+^/A2B5^+^, showing that they didn't differentiate into mature cells in differentiation medium (Figure 6C). RT-PCR proved knockdown of STIM1 and Orai1 in most cells treated with siRNA of STIM1, Orai1, or both of them (Figure S4D). Taken together, these results suggest that STIM1 or Orai as SOCE channel does not have a significant role in the induction of neuronal differentiation from A2B5^+^NPCs unlike TRPC5.

### Conclusion

Our data suggest that the amplitudes of SOCE are increased in differentiated neuronal cells from A2B5^+^ NPCs and the up-regulation of TRPC5 plays a major role in the change of SOCE in the neuronal differentiation from A2B5^+^ NPCs. Further studies are needed to clarify the signal transduction and their regulation of TRPC-mediated Ca^2+^ mechanism in the neuronal differentiation from NPCs regardless of the developmental brain or adult brain to enhance understanding the mechanism of neuronal differentiation from NPCs and for the potential therapeutic applications. We also interested in the downstream effects of SOCE on the transcriptional regulation of key genes involved in neurodevelopment in this system or examine these experiments in embryonic rat brain, to confirm that TRPC5 has the same influence.

## Supporting Information

Table S1 Oligonucleotide primers used for PCR. Abbreviations: TRPC, canonical transient receptor potential channel; MAP2, Microtubule-associated protein 2; Orai, calcium release-activated calcium modulator; STIM, stromal interaction molecule.
(0.04 MB DOC)

Table S2 siRNA sequences for TRPC5 and TRPC6. Abbreviations: siRNA; small interfering RNA, TRPC, canonical transient receptor potential channel.
(0.03 MB DOC)

Table S3 Differential expression of Orai subtype and STIM1 between differentiated cells and A2B5 + NPCs. Abbreviations: Orai, calcium release-activated calcium modulator; STIM, stromal interaction molecule.
(0.03 MB DOC)

Figure S1 Effect of membrane depolarization on TG-stimulated Ca2+-entry in A2B5 +NPCs versus differentiated neuronal cells. TG-stimulated Ba2+ influx experiments similar to those described in Fig. 3 were performed in Ca2+ free HBSS containing either normal KCl or high KCl (133 mM KCl). Differentiated neuronal cells show higher amplitude of SOCE than A2B5+ NPCs in absence or presence of high KCl. The term indicated as the amplitude of SOCE is calculated as (Ratio after treating Ba2+) - (Ratio before treating Ba2+).*, P < 0.01 compared with corresponding A2B5+ NPCs in absence or presence of high KCl.
(0.48 MB TIF)

Figure S2 Effect of RR on TG-stimulated Ca2+-influex and cell viability in differentiated neuronal cells. TG-stimulated Ba2+ influx experiments were described in Fig. 3. After store depletion, TG-stimulated Ba2+ influx was inhibited by the addition of RR (20 µM). Each trace is the average of differentiated neuronal cells. The time of Ba2+ addition is indicated by the dark grey and that of 2-APB is done by the grey. Error bars represent SE. The data are representative of at least 3 separate experiments.
(0.50 MB TIF)

Figure S3 Expression of mRNA of TRPC subtypes in A2B5+ NPCs. (A): Expression of mRNA for TRPC 1-7 was confirmed in the A2B5+ cells, and cerebrum was used as control. RNA was extracted from A2B5+ NPCs, reverse-transcribed, and subjected to RT-PCR using primers specific for each TRPC subtypes. PCR products of the expected size are seen for TRPC. (B): Expression of TRPCs in proliferating or differentiated A2B5 cells.
(0.99 MB TIF)

Figure S4 Effect of siRNA targeting TRPC5 and TRPC6 on neuronal differentiation of NPCs in differentiation conditions. (A-C): A2B5+ NPCs treated with siRNA of Orai1 and STIM1 in differentiation condition developed cellular processes and differentiated into neuronal cells (MAP2+/Tuj1+), astrocytes (GFAP+) and oligodendrocytes (O4+) like control cells. (D): RT-PCR proved knockdown of STIM1 and Orai1 in most cells treated with siRNA of STIM1, Orai1, or both of them. Scale bars, 50µm.
(5.73 MB TIF)
