# Supplementary material for: A Role of Canonical Transient Receptor Potential 5 Channel in Neuronal Differentiation from A2B5 Neural Progenitor Cells
Source: PLoS One. 2010 May 7;5(5):e10359. doi: 10.1371/journal.pone.0010359 (PMC2866321; doi:10.1371/journal.pone.0010359)
Supplement: Table S1 — Oligonucleotide primers used for PCR. Abbreviations: TRPC, canonical transient receptor potential channel; MAP2, Microtubule-associated protein 2; Orai, calcium release-activated calcium modulator; STIM, stromal interaction molecule. (0.04 MB DOC) [file pone.0010359.s001.doc]

**Supplemental table 1. Oligonucleotide primers used for PCR**

| **Primer** | **Forward** | **Reverse** |
| --- | --- | --- |
| **TRPC1** | **GGC TGT GAT TAT CGG AAC TTA C** | **TGC ACT AGG CAG CAC ATC AC** |
| **TRPC2** | **CAG TTC CTG GTG CCT GAG TT** | **AGC ATC GTC CTC GAT CTT CTG** |
| **TRPC3** | **CAT TGG CTA TGT CCT TTA TGG AAT A** | **AGA CTG AAG GGT GGA GGT AAT G** |
| **TRPC4** | **AAG ACA CTG GGC GGT GAA GA** | **TGT AAT CCT GAA GTC CAC CAT** |
| **TRPC5** | **ACG GAC TGA ACC AGC TTT ATT** | **GTT GTA TGT CCC AAA CAT AGT AGC T** |
| **TRPC6** | **TCA ACC TCT ACT CCT ACT ACA TTG GT** | **TTA TAG ACT CCA TAC AGA ACA TAG CCT** |
| **TRPC7** | **GCA TGT TGT CCG TCT TCG T** | **CCT GGC GTA GAA GTA TGC** |
| **MAP2** | **GCC ATG ATC TTT CCC CTC TGG CTT** | **GTC TGG TTT TAC GGG TTG GCT GTC** |
| **NESTIN** | **CTA CCA GGA GCG CGT GGC** | **TCC ACA GCC AGC AAC TT** |
| **STIM1** | **CAGTGAAACACAGCACCTTCC** | **AAGAGCACTGTATCCAGAGCC** |
| **Orai1** | **TCGGTCAAGGAGTCCCCCCAT** | **GTCCTGTAAGCGGGCAAACTC** |
| **Orai2** | **GCTGAGCTTAACGTGCCTATC** | **GGAGTTCAGGTTGTGGATGTT** |
| **Orai3** | **CCCTTAGTCCAGCTTCCAATC** | **CCAAGGAGCGGTAGAAATGCA** |
| **Actin** | **AAG ATC CRG ACC GAG CGT GG** | **CAG CAC TGT GTT GGC ATA GAG G** |

Abbreviations:TRPC, canonical transient receptor potential channel; MAP2, Microtubule-associated protein 2; Orai, calcium release-activated calcium modulator; STIM, stromal interaction molecule.
