# Supplementary material for: A Role of Canonical Transient Receptor Potential 5 Channel in Neuronal Differentiation from A2B5 Neural Progenitor Cells
Source: PLoS One. 2010 May 7;5(5):e10359. doi: 10.1371/journal.pone.0010359 (PMC2866321; doi:10.1371/journal.pone.0010359)
Supplement: Table S2 — siRNA sequences for TRPC5 and TRPC6. Abbreviations: siRNA; small interfering RNA, TRPC, canonical transient receptor potential channel. (0.03 MB DOC) [file pone.0010359.s002.doc]

**Supplemental table 2. siRNA sequences for TRPC5 and TRPC6**

| **siRNA** | **Sense** | **Antisense** |
| --- | --- | --- |
| **TRPC5** | **CAC UCU UCG CGA UAU CGA A** | **UUC GAU AUC GCG AAG AGU G** |
| **TRPC5** | **CAG ACA ACA CAA AUC UGA A** | **UUC AGA UUU GUG UUG UCU G′** |
| **TRPC5** | **GAC AUC AGA UAU UCU CAG A** | **UCU GAG AAU AUC UGA UGU C** |
| **TRPC6** | **CAC UCA ACC AGC CUG UCU A′** | **UAG ACA GGC UGG UUG AGU G** |
| **TRPC6** | **CAC UCU AGA UCC AGG AUC** | **UGA UCC UGG AUC UAG AGU G** |
| **TRPC6** | **CAU CAU UCA UUG CAA GUA U** | **AAU CUU GCA AUG AAU GAU G** |

Abbreviations: siRNA; small interfering RNA, TRPC, canonical transient receptor potential channel;
