# Supplementary material for: A Role of Canonical Transient Receptor Potential 5 Channel in Neuronal Differentiation from A2B5 Neural Progenitor Cells
Source: PLoS One. 2010 May 7;5(5):e10359. doi: 10.1371/journal.pone.0010359 (PMC2866321; doi:10.1371/journal.pone.0010359)
Supplement: Table S3 — Differential expression of Orai subtype and STIM1 between differentiated cells and A2B5 + NPCs. Abbreviations: Orai, calcium release-activated calcium modulator; STIM, stromal interaction molecule. (0.03 MB DOC) [file pone.0010359.s003.doc]

**Supplementary table 3. Differential expression of Orai subtype and STIM1 between differentiated cells and A2B5 ^+^ NPCs**

| **Subtypes** | **differential expression ratio of mRNA** | **TaqMan assay** |
| --- | --- | --- |
| Orai1 | **2.5** | **Rn 20397170_m1** |
| Orai2 | **1.8** | **Rn01480748_m1** |
| Orai3 | **1.7** | **Rn01774170_m1** |
| STIM1 | **1.5** | **Rn 01506496 _m1** |

Abbreviations: Orai, calcium release-activated calcium modulator; STIM, stromal interaction molecule.
