# Supplementary figures and images for: A Role of Canonical Transient Receptor Potential 5 Channel in Neuronal Differentiation from A2B5 Neural Progenitor Cells
Source: PLoS One. 2010 May 7;5(5):e10359. doi: 10.1371/journal.pone.0010359 (PMC2866321; doi:10.1371/journal.pone.0010359)

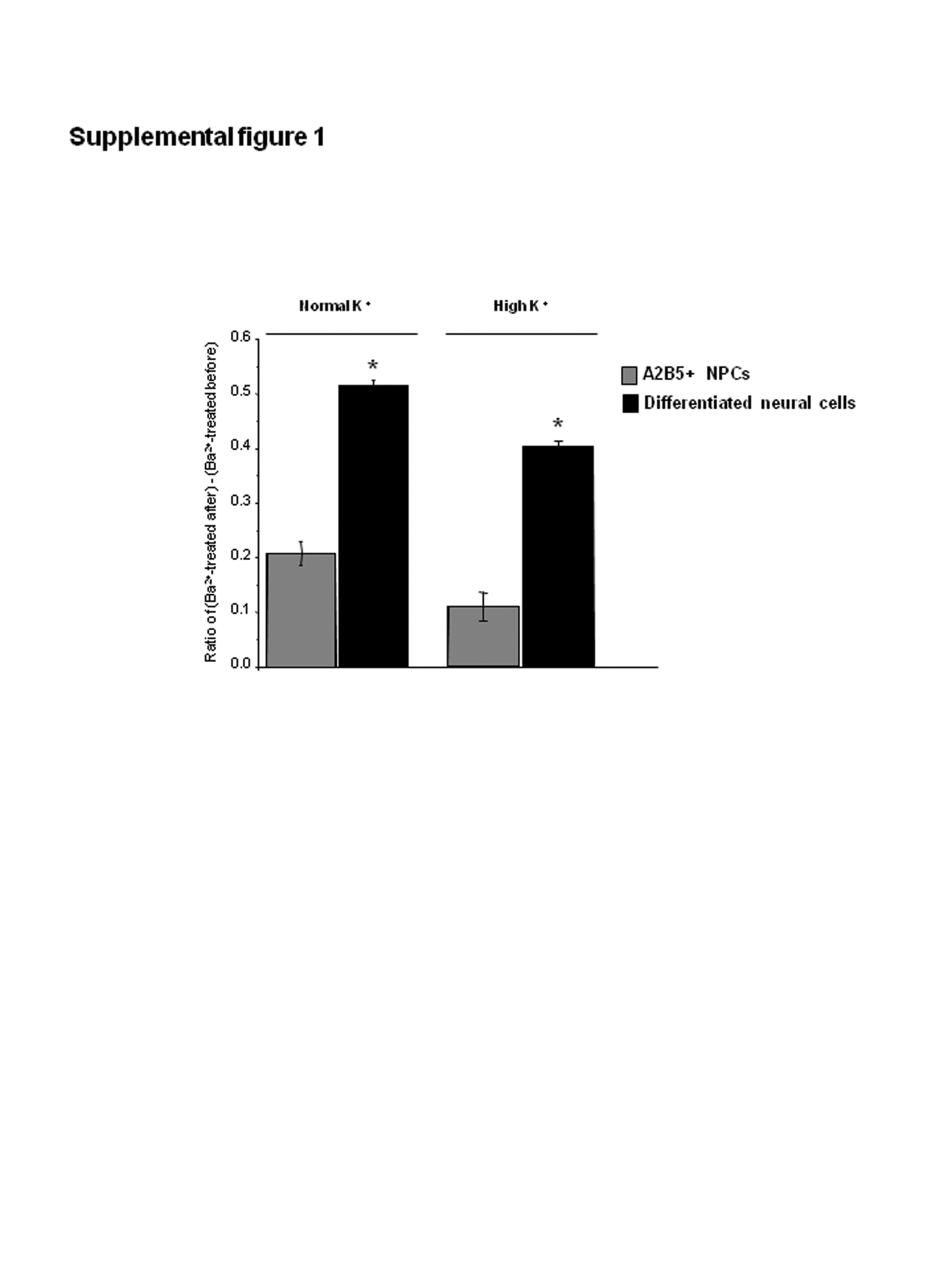

Supplement: Figure S1 — Effect of membrane depolarization on TG-stimulated Ca2+-entry in A2B5 +NPCs versus differentiated neuronal cells. TG-stimulated Ba2+ influx experiments similar to those described in Fig. 3 were performed in Ca2+ free HBSS containing either normal KCl or high KCl (133 mM KCl). Differentiated neuronal cells show higher amplitude of SOCE than A2B5+ NPCs in absence or presence of high KCl. The term indicated as the amplitude of SOCE is calculated as (Ratio after treating Ba2+) - (Ratio before treating Ba2+).*, P < 0.01 compared with corresponding A2B5+ NPCs in absence or presence of high KCl. (0.48 MB TIF) [file pone.0010359.s004.tif]

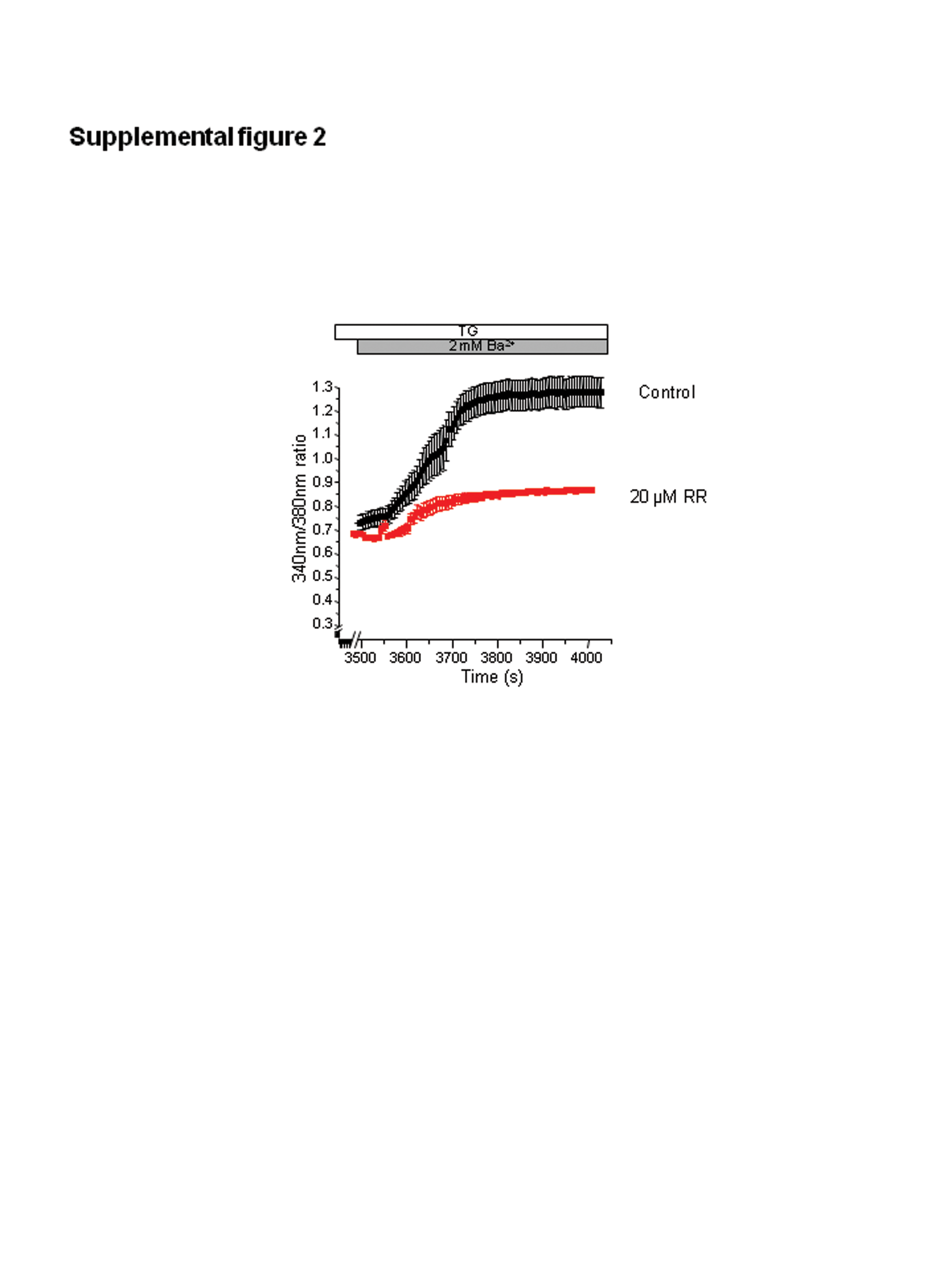

Supplement: Figure S2 — Effect of RR on TG-stimulated Ca2+-influex and cell viability in differentiated neuronal cells. TG-stimulated Ba2+ influx experiments were described in Fig. 3. After store depletion, TG-stimulated Ba2+ influx was inhibited by the addition of RR (20 µM). Each trace is the average of differentiated neuronal cells. The time of Ba2+ addition is indicated by the dark grey and that of 2-APB is done by the grey. Error bars represent SE. The data are representative of at least 3 separate experiments. (0.50 MB TIF) [file pone.0010359.s005.tif]

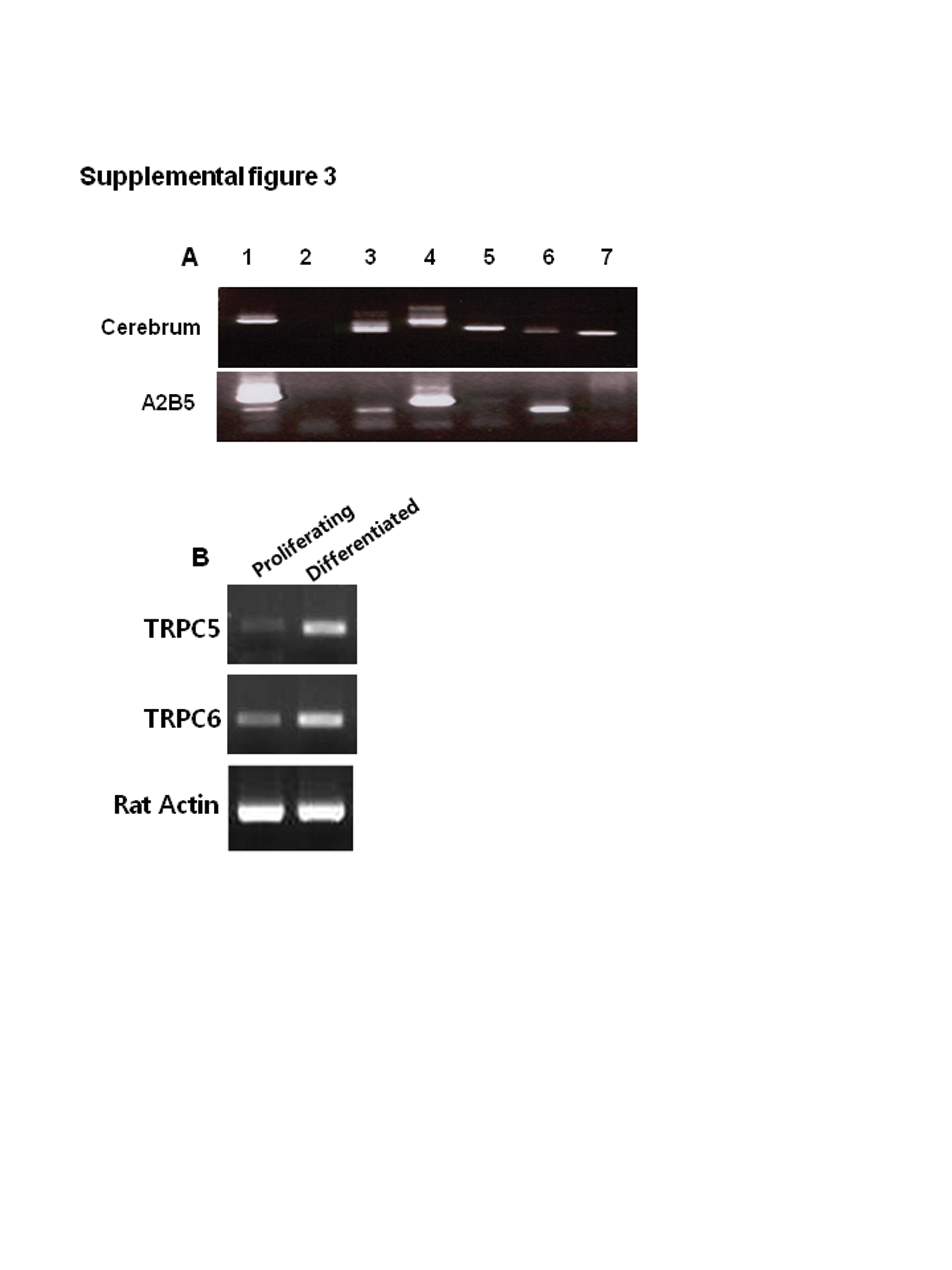

Supplement: Figure S3 — Expression of mRNA of TRPC subtypes in A2B5+ NPCs. (A): Expression of mRNA for TRPC 1-7 was confirmed in the A2B5+ cells, and cerebrum was used as control. RNA was extracted from A2B5+ NPCs, reverse-transcribed, and subjected to RT-PCR using primers specific for each TRPC subtypes. PCR products of the expected size are seen for TRPC. (B): Expression of TRPCs in proliferating or differentiated A2B5 cells. (0.99 MB TIF) [file pone.0010359.s006.tif]

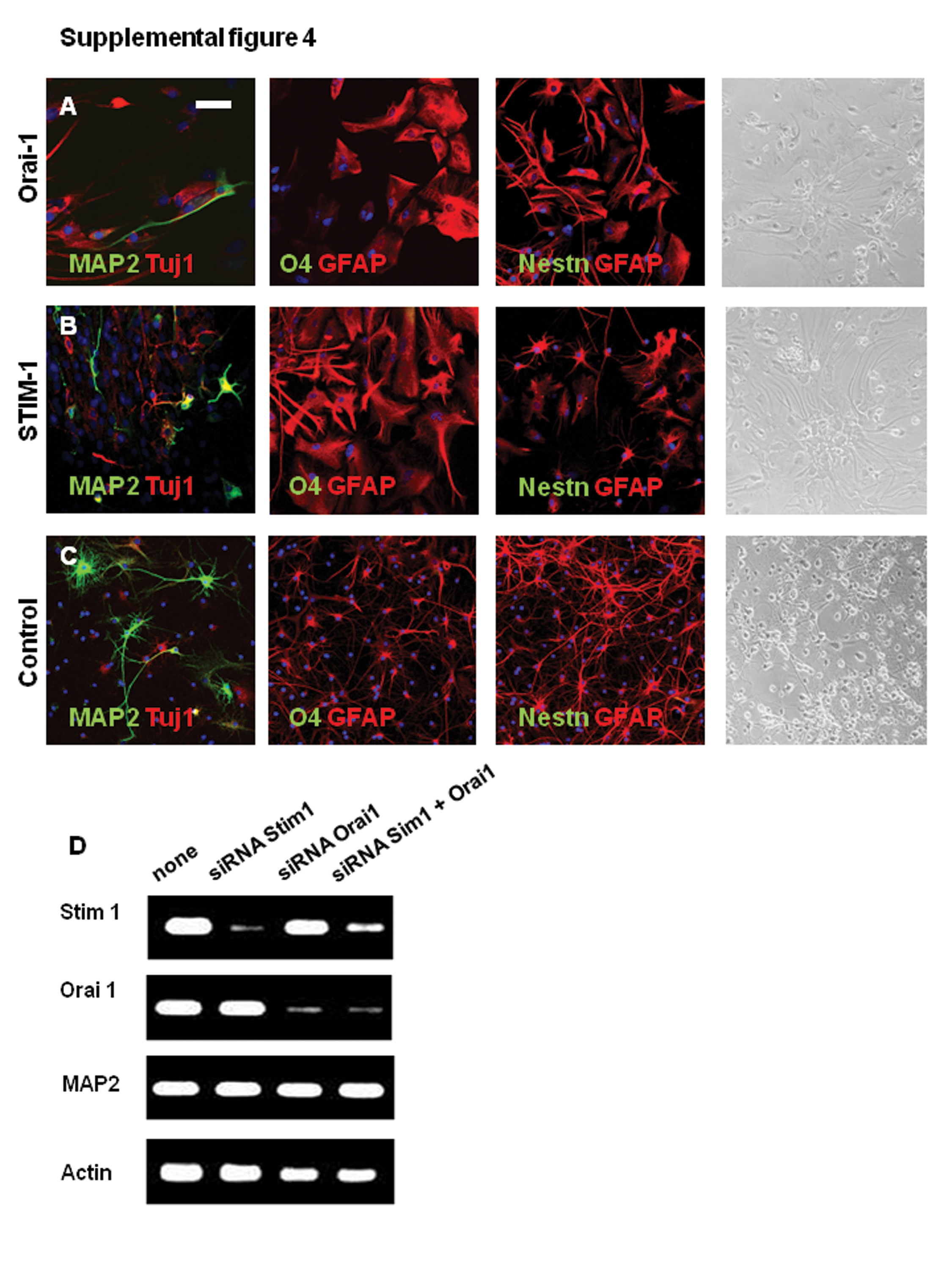

Supplement: Figure S4 — Effect of siRNA targeting TRPC5 and TRPC6 on neuronal differentiation of NPCs in differentiation conditions. (A-C): A2B5+ NPCs treated with siRNA of Orai1 and STIM1 in differentiation condition developed cellular processes and differentiated into neuronal cells (MAP2+/Tuj1+), astrocytes (GFAP+) and oligodendrocytes (O4+) like control cells. (D): RT-PCR proved knockdown of STIM1 and Orai1 in most cells treated with siRNA of STIM1, Orai1, or both of them. Scale bars, 50µm. (5.73 MB TIF) [file pone.0010359.s007.tif]
